# Fitness Effects of Thermal Stress Differ Between Outcrossing and Selfing Populations in *Caenorhabditis elegans*

**DOI:** 10.1007/s11692-017-9413-z

**Published:** 2017-03-03

**Authors:** Agata Plesnar-Bielak, Marta K. Labocha, Paulina Kosztyła, Katarzyna R. Woch, Weronika M. Banot, Karolina Sychta, Magdalena Skarboń, Monika A. Prus, Zofia M. Prokop

**Affiliations:** Institute of Environmental Sciences, Jagiellonian University, Gronostajowa 7, 30-387 Kraków, Poland

**Keywords:** Mating systems, Outcrossing, Temperature, Adaptation, Fitness, Stress

## Abstract

The maintenance of males and outcrossing is widespread, despite considerable costs of males. By enabling recombination between distinct genotypes, outcrossing may be advantageous during adaptation to novel environments and if so, it should be selected for under environmental challenge. However, a given environmental change may influence fitness of male, female, and hermaphrodite or asexual individuals differently, and hence the relationship between reproductive system and dynamics of adaptation to novel conditions may not be driven solely by the level of outcrossing and recombination. This has important implications for studies investigating the evolution of reproductive modes in the context of environmental changes, and for the extent to which their findings can be generalized. Here, we use *Caenorhabditis elegans*—a free-living nematode species in which hermaphrodites (capable of selfing but not cross-fertilizing each other) coexist with males (capable of fertilizing hermaphrodites)—to investigate the response of wild type as well as obligatorily outcrossing and obligatorily selfing lines to stressfully increased ambient temperature. We found that thermal stress affects fitness of outcrossers much more drastically than that of selfers. This shows that apart from the potential for recombination, the selective pressures imposed by the same environmental change can differ between populations expressing different reproductive systems and affect their adaptive potential.

## Introduction

Outcrossing, *i.e*. reproduction by fusing gametes of distinct individuals, remains one of evolution’s mysteries. Compared to uniparental reproduction (asexuality or self-fertilization), it incurs considerable costs, particularly when associated with the production of males which facilitate outcrossing but do not themselves bear offspring, while requiring energy resources that could have been used otherwise (Maynard Smith 1971, 1978; Lloyd 1980; Bell 1982; Uyenoyama 1984; Lively and Lloyd 1990; Anderson et al. 2010). Nevertheless, a vast majority of animal species produce males, suggesting that this mode of reproduction does bring some significant selective advantages. Most theoretical explanations proposed to date relate to the role of recombination. Outcrossing shuffles genes among individuals, creating new combinations of alleles. Therefore, it can break apart selection interference between beneficial and deleterious mutations (Hill-Robertson effect), facilitating the spread of the former and the purging of the latter (reviewed by Otto 2009). Importantly, this may also lead to (some of) the offspring of outcrossing individuals having increased fitness in a changing environment (Stebbins 1957). Both these factors can accelerate the rate of adaptation to novel environmental conditions. Thus, the benefits of outcrossing should be particularly pronounced under environmental change (e.g. Colegrave 2002; Goddard et al. 2005; Morran et al. 2009a, b; but see; Zeyl and Bell 1997).

However, the same change in the external environment may impose different levels of stress on individuals and populations differing in breeding system. If this is the case, then aside the recombination rate, also the strength of selection may differ between such populations (Parsons 1987; Kondrashov and Houle 1994; Jasnos et al. 2008, but see; Agrawal and Whitlock 2010), contributing to differences in adaptation process. Furthermore, these effects may be specific to the type of environmental change experienced by the populations.

This has important implications for investigating the role of recombination in adapting to environmental change. For example, if a novel environment applied in a study imposes stronger selection on outcrossing populations (compared with selfing or asexual ones), leading to faster evolutionary response, and this difference in selective pressures is then neglected when interpreting the results, the higher adaptation rate may be attributed primarily to the effects of genetic shuffling. In consequence, generalizing the effects of such studies may lead to overestimating the recombination’s impact on adaptation. The reverse scenario may be true if the novel selective pressure is stronger on selfers or asexuals. Thus, when investigating the role of outcrossing in adaptation to novel conditions, it is important to understand how these conditions influence fitness of individuals expressing particular reproductive strategies.

Many of the studies investigating the problem of male maintenance associated with the existence of outcrossing have used *Caenorhabditis elegans*, a common model species in evolutionary and genetic research (Gray and Cutter 2014). *C. elegans* is androdioecious, with hermaphrodites coexisting with males. Hermaphrodites are capable of both selfing and outcrossing with males, but they are not able to mate with other hermaphrodites (outcrossing occurs only through mating with males). Sex is determined by the ratio of X chromosomes to autosomes (Hodgkin 1987) with hermaphrodites having AA:XX genotype and males AA:X0. Hence, a male forms when a gamete carrying one X fuses with a gamete carrying no sex chromosome—which can happen either through self-fertilization following X-chromosomes nondisjunction during meiosis, or via outcrossing (since half of the gametes produced by males lack the X chromosome).

Selfing is a predominant reproductive mode in *C. elegans*. Male frequencies vary between strains (reviewed in Anderson et al. 2010). However, in most populations (including the well-studied laboratory strain N2) they are very low, often similar to those of nondisjunction events (Hodgkin 1983; Chasnov and Chow 2002; Teotònio et al. 2006, but see Wegewitz et al. 2008). Adult males and hermaphrodites show no difference in viability (Hodgkin 1987; Gems and Ridddle 1996, 2000). Although males survive dauer (an alternative developmental stage induced by stressful conditions) slightly better than hermaphrodites (Morran et al. 2009a, b), this difference is very small. Male fertilization success depends on their frequency in a population, being the highest when the proportion of males is 0.2 (Stewart and Phillips 2002). In the N2 laboratory strain males may sire 70% of the offspring produced in such populations (Stewart and Phillips 2002). However, these rates are still too low to prevent a gradual loss of males from populations. Moreover, as inbreeding depression has not been recorded in the species (Johnson and Wood 1982; Johnson and Hutchinson 1993; Chasnov and Chow 2002; Dolgin et al. 2007), suggesting that prolonged inbreeding has purged mutation load, offspring resulting from outcrossing is not predicted to be fitter than offspring of selfing hermaphrodites (cf. Anderson et al. 2010). Altogether, this suggests that males should be easily lost from populations—which is supported by the results of experiments performed under standard laboratory conditions (Stewart and Phillips 2002; Chasnov and Chow 2002; Cutter et al. 2003; Cutter 2005)—and that they do not play important role in *C. elegans* evolution.

However, the fact that a large fraction of the genome is devoted to male functions (Jiang et al. 2001) and that genes expressed only in males are among the most conserved in this species (Cutter 2005) questions such reasoning. Unless *C. elegans* has become predominantly selfing only recently, male-specific genes must have been maintained and conserved by selection acting on males (Loewe and Cutter 2008). This suggests that outcrossing or/and males as such have fitness advantage in at least some conditions and circumstances. Indeed, the hypothesis that outcrossing becomes favorable in populations adapting to environmental challenge has been gaining support over the last several years (Morran et al. 2009a, b, 2013; Teotònio et al. 2012; Lopes et al. 2008; Carvalho et al. 2014; but see; Theologidis et al. 2014).

Extensive knowledge about the genetics of *C. elegans* allows manipulating its mating system, providing a useful tool for experimental tests of the role of outcrossing in adaptation. Scientists have identified several mutations altering dynamics of mating systems in this species (see Anderson et al. 2010 for review), with mutations in *fog-2* and *xol-1* genes being among the most frequently used in evolutionary studies (Stewart and Phillips 2002; Katju et al. 2008; Morran et al. 2009a, b). The first of those genes, *fog-2*, produces a protein inhibiting production of sperm in hermaphrodites homozygous for this locus (Schedl and Kimble 1988; Clifford et al. 2000; Nayak et al. 2005). Thus, this mutation effectively turns hermaphrodites into females, enforcing obligate outcrossing in a mutant population. Mutation in *xol-1* gene causes obligate selfing, as it disturbs dosage compensation rendering males inviable (Miller et al. 1988; Rhind et al. 1995). The possibility to utilize the above mutations enables establishment of populations differing in mating systems. This makes *C. elegans* a species in which the hypotheses considering male maintenance and the role of outcrossing can be precisely tested.

**Fig. 1:**
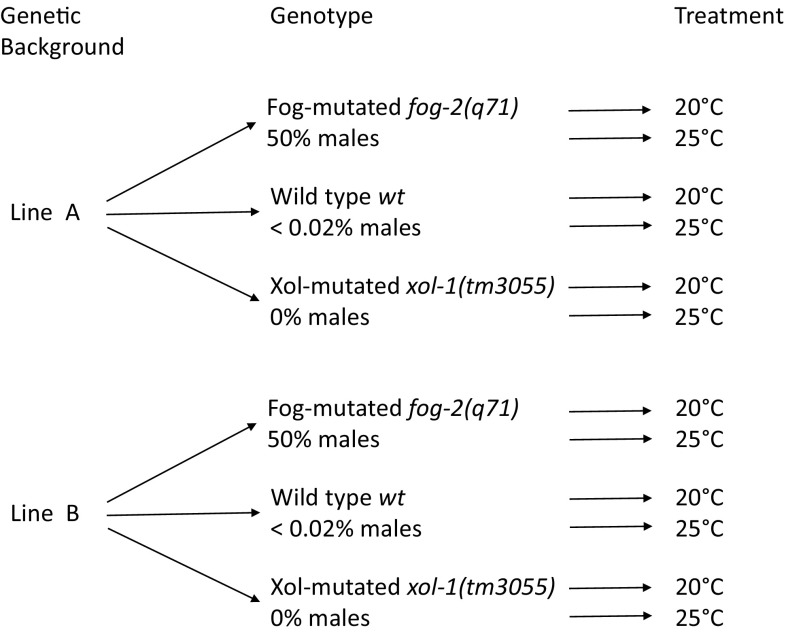
Schematic representation of our experimental design

However, as mentioned above, the same change in external environment may affect fitness of males, females, and hermaphrodites differently, hence imposing disparate selective pressures on different mutants and reproductive systems. Here, we use replicated lines derived from the N2 strain to investigate the effects of a stressful novel environment (increased ambient temperature) on fitness of *fog-2* (obligatorily outcrossing) and *xol-1* (obligatorily selfing) mutants, as well as wild type, of *C. elegans* (Fig. 1).

## Materials and Methods

### Animal Culture

We followed standard procedures for culturing and manipulation of *C. elegans* (Brenner 1974). Animals were grown on 6 cm Petri dishes with standard Nematode growth medium (NGM) seeded with 200 µl of OP50 strain of *Escherichia coli* (Stiernagle 2006).

We used a wild type N2 (Bristol) strain of *C. elegans*, obtained from the *Caenorhabditis* Genetics Center (CGC, University of Minnesota, USA). In this strain, the frequency of males is approximately 0.002 (Hodkin et al. 1983; Chasnov and Chow 2002; Teotònio et al. 2006), which does not exceed the rate at which they are produced by spontaneous non-disjunction events (Hodgkin et al. 1979; Rose and Baillie 1979; Teotònio et al. 2006). Two independent isolines (henceforth referred to as source lines A and B) were established by 20 generations of single hermaphrodite transfer.

### Mating System Manipulations

Two reproductive system-altering mutations were independently introgressed into each of the two source lines in order to obtain two sets of obligatorily selfing, obligatorily outcrossing, and wild type (facultatively outcrossing) populations with otherwise similar genetic backgrounds (Fig. 1).

To obtain obligatorily selfing lines, *xol-1* mutation *tm3055* was introgressed into each of the source lines (carrying the wild type *xol-1* allele, henceforth: *wt*), according to a modified protocol described by Theologidis and colleagues (2014). (1) Hermaphrodites from strain TY1807 homozygous for the *tm3055* mutation were placed on Petri dishes with an excess of source line (*wt*/*wt*) males (P generation). (2) The F1 hermaphrodite offspring were individually isolated and allowed to reproduce by self-fertilization for 1 day, after which they were genotyped to confirm *tm3055*/*wt* heterozygosity (this step was necessary since *C. elegans* hermaphrodites can reproduce by self-fertilization regardless of the presence of males, which in this case would have resulted in *tm3055*/ *tm3055* offspring). (3) Hermaphrodite offspring (F2) of the verified heterozygotes were individually placed on Petri dishes and an excess of source line (*wt*/*wt*) males was added to each dish. (4) Their offspring (F3) were screened for the presence of males once they reached the L4 stage; the dishes containing males were discarded. The absence of males indicated that the maternal (F2) hermaphrodite was homozygous for the *tm3055* allele, making all male offspring inviable (5) From the dishes containing no males, F3 hermaphrodites were individually isolated to Petri dishes and allowed to reproduce by self-fertilization for 1 day, after which they were genotyped to confirm *tm3055*/*wt* heterozygosity (see step (2)). Steps 2–5 were repeated eight times in total.

To obtain obligatorily outcrossing lines, *fog-2* mutation *q71* was introduced into each of the *wt* source lines (carrying the wild type *fog-2* allele), using a protocol described by Teotònio et al. (2012). Parental hermaphrodites from a given source line (*wt*/*wt*) were mated with males from the JK574 strain homozygous for the *q71* mutation, and their hermaphrodite offspring (*q71*/*wt* originating from mating with males and *wt*/*wt* originating from self-fertilization of hermaphrodites) were separately selfed to generate F2. Twenty F2 hermaphrodites from each of the lines were picked onto individual plates and let to self. F3 progeny was checked for phenotype “piano” (accumulation of unfertilized oocytes in the gonads) and absence of F4 progeny, which indicated homozygosity for *fog-2* mutation *q71* in parental hermaphrodite. There were 8 other such cycles of introgression, starting with the F3 fog-females being mated with an excess of males from the source lines.

Wild-type source lines were subjected to single hermaphrodite transfer for the time necessary to complete eight cycles of introgression in the mutant lines, before being used in the fitness assays, so the inbreeding in all lines was similar. After completing the introgression (or single transfers in wild type lines) all the lines were frozen at −80 °C.

### Fitness Assay

All the lines were thawed and placed on Petri dishes at 20 °C. After 4 days (one generation) of acclimatization at 20 °C, the lines were synchronized and cleared of any contaminations using bleaching (Stiernagle 2006; briefly: the procedure involves treating the animals with hypochlorite solution which dissolves adults and larvae but leaves the eggs, which are protected by shells, intact). After bleaching, for each source line × breeding system combination, approximately 2000 eggs were transferred on two 14 cm diameter Petri dishes (1000 eggs per dish), one of which was subsequently placed at 20 °C, and the other at 25 °C. The temperature error range of the incubators was 0.5 °C. The worms were left at the experimental temperatures for two generations before the fitness assay was performed. High population densities were prevented by chunking procedure, *i.e*., each generation, a small piece (approx. 1 cm^2^) of agar containing worms was carved and transferred onto a new 14 cm diameter Petri dish seeded with bacteria medium. While the numbers of individuals transferred in this manner are likely to differ between populations, they were consistently small enough, compared to dish size, as to ensure *ad libitum* space and food access (as confirmed by the excess of bacteria present on old plates when the chunks were carved), thus minimizing the risk of any density-dependent effects.

After the two generations of acclimation, for each source line × breeding system × temperature combination, 15 hermaphrodites (for wild type and *xol-*mutated populations) or 15 pairs (for *fog-*mutated populations) in the last larval stage (L4) were individually transferred to 6 cm Petri dishes. Each dish was then returned to its respective temperature. After 24 h, we transferred the animals onto new plates, while the dishes with eggs were left for 2 days, until the offspring reached L3/L4 larval stage. We repeated this procedure (transferring adult individuals onto new plates while leaving the eggs they had laid since the previous transfer for further development) for 7 days. At the L3/L4 stage, the offspring were counted, and each scored worm was aspired out with a vacuum pump to prevent counting the same individual multiple times. Each dish was re-inspected the next day in order to score the offspring which were overlooked during the first counting (e.g. because they crawled under the agar or on the side of the dish). The total number of offspring (*i.e*., the lifetime reproductive success) of each experimental hermaphrodite/pair was then summed up over all days it had reproduced. We eliminated from the analysis data obtained from individuals which could not be found on the plates and therefore we could not be certain when they finished reproducing.

### Statistical Analyses

Data were analyzed using R.3.2.0 (R Core Team 2015).

Proportions of infertile individuals/pairs were analyzed using Fisher exact test for each of the source lines separately at each temperature. Hence, four analyses were performed, comparing infertility rates across breeding systems (analysis for the source line A at 20 °C, analysis for the source line B at 20 °C, analysis for the source line A at 25 °C, analysis for the source line A at 25 °C). Additionally, we compared infertility rates across temperatures applying Fisher exact tests within each mating system and source line combination.

Data on lifetime reproductive success showed high heterogeneity of variance. Thus, they were analyzed using the gls function, implemented in the nlme package (Pinheiro et al. 2014), which allows to build models with differing variance structures in the data (Davidian and Giltinan 1995). Temperature, breeding system and source line (and all interactions) were included as fixed effects and the number of offspring was a response variable. First, we fitted a standard ANOVA with homogenous variance structure, and visually inspected model residuals plotted against factor levels. Based on these plots, in order to choose the optimal variance structure, we fitted four further models: (i) a model allowing for differences in variances among breeding systems, (ii) a model allowing for differences in variances between temperatures, (iii) a model allowing for different variances among all combinations of breeding system × temperature, and (iv) a model allowing for different variances among all combinations of breeding system × temperature × source line. VarIdent variance structure was used in these models, allowing for differences in variance among levels of nominal variables (Zuur et al. 2009). Each of the models i–iv was then compared to the standard ANOVA model using log-likelihood ratio test, which showed that they were all significantly better than the standard model. Thus, the four models were ranked using AIC criterion (we could not perform log-likelihood ratio test as these models are not nested). Model iv had the lowest AIC score and hence it is reported in the Results section.

## Results

### Infertility Rates

At 20 °C, infertility rates were low and did not differ between the breeding systems (Fisher’s exact tests, source line A: p = 0.096, source line B: p = 0.343). Two out of 14 assayed pairs were infertile in the *fog*-mutated line A and three out of fifteen assayed pairs were infertile in the *fog*-mutated line B. There were no infertile individuals in the *xol*-mutated lines. In the wild type lines, 0 and 2 individuals failed to lay eggs in lines A and B, respectively, out of 15 assayed in each line. In contrast, at increased temperature, the rates of infertility were strikingly high in the *fog*-mutated lines; 10 and 7 out of 15 tested pairs were infertile in the *fog*-mutated lines compared to 2 and 0 out of 15 in the *xol*-mutated lines and 1 and 0 out of 15 in wild type lines (lines A and B, respectively; Fisher’s exact test, p < 0.001 for both line A and line B).

Fertility rates decreased significantly with temperature in the *fog*-mutated line A (Fisher’s exact test, p = 0.008). The *fog*-mutated line B also tended to show reduction in fertility rate, although the trend was not significant (p = 0.109). Fertility did not vary between temperatures in the *xol*-mutated lines (Fisher’s exact test, line A: p = 0.483, line B: p = 1) or the wild type lines (Fisher’s exact test, line A: p = 1, line B: p = 0.483).

### Lifetime Reproductive Success

**Fig. 2:**
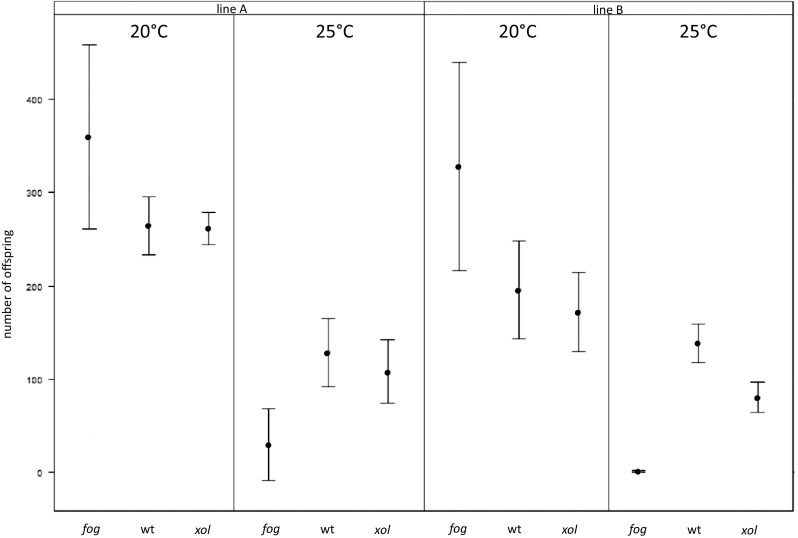
Least square means and confidence intervals for the number of offspring produced by the obligatorily selfing (*fog-*mutated—‘*fog’*), facultatively outcrossing (wild type—‘wt’) and obligatorily outcrossing (*xol-*mutated—‘*xol*’) lines for each of the source lines and thermal treatments. The estimates were calculated from the linear model allowing for different variances among breeding systems, temperatures and source lines

Lifetime reproductive success was affected by interactions between breeding system and temperature and, less strongly, between line and temperature, but not by the breeding system × line or the three-way interaction (Table 1; Fig. 2), which were hence removed from the final model. In 20 °C, *fog-*mutated lines had higher reproductive success than wild type (p = 0.005) and *xol-*mutated (p = 0.002) lines, whereas in 25 °C the situation was reversed (both p < 0.001) (Fig. 2), which was largely due to the high levels of infertility in *fog-*mutated lines (see above).

**Table 1:** Effects of temperature, breeding and source line together with and all the interactions on lifetime reproductive success (number of offspring produced) of *C. elegans* hermaphrodites (*xol*-mutated and wild type lines) and pairs of males and females (*fog*-mutated lines) analyzed using linear model with differences in variances among breeding systems, temperatures and source lines

Effect	df.	F	p
Temperature	1;167	1392.749	<0.001
Breeding system	2;167	160.915	<0.001
Source line	1;167	14.151	<0.001
Temperature x breeding system	2;167	16.352	<0.001
Temperature x source line	1;167	8.634	0.004
Breeding system x source line	2;167	1.119	0.329
Temperature x breeding system x source line	2;167	0.394	0.675
Error	167	–	–

## Discussion

Our study reveals the reduction of reproductive success at high temperature in all three breeding systems. This is in line with previous studies demonstrating that *C. elegans* fecundity is highest at 20 °C and declines with increasing temperature (e.g. Byerly et al. 1976; McMullen et al. 2012; Petrella 2014, but see Zhang et al. 2015). The decrease of reproductive function at high temperatures seems to be associated with functioning of both spermathogenic and oogenic germ lines (Aprison and Ruvinsky 2014; Petrella 2014), although the relative contribution of these two factors varies between strains (Petrella 2014).

At 20 °C, the outcrossing (*fog*-mutated) pairs produced more offspring than selfing hermaphrodites from both wild type and *xol*-mutated lines. It is likely that inhibition of sperm production resulted in redirecting more resources into egg production so that a fraction of germ cells that could not differentiate as sperm developed as oocytes (Schedl and Kimble 1988). A similar pattern has been observed by Theologidis and colleagues (2014), who have found fecundity of *fog*-mutated females to be higher than hermaphrodite fecundity at high salinity (although this effect has not translated into population level).

More interestingly, the influence of temperature on reproductive success was much more dramatic in the *fog*-mutated lines compared to both *xol*-mutated and wild type selfing lines (Fig. 2). In particular, the proportion of pairs which did not produce any eggs rose from 0.13 to 0.14 to 0.67 and 0.47 in the *fog*-mutated lines. This decrease of fertility was only significant in one of the lines; however, in the other one the clear trend in the same direction was coupled with extremely low numbers of offspring produced by fertile pairs (only one or two offspring produced by the fertile pairs). In contrast, in selfing lines, while the offspring number declined with increased temperature, the infertility rates remained low (*xol*-mutated: 0.13 and 0.07, wild type: 0.07 and 0).

Such drastic fitness decline in outcrossers under thermal stress could not be due to elevated mortality in *fog*-mutated lines as most individuals either remained alive for at least 1 day (usually longer) after they finished reproduction or had never started laying eggs. Only two females in each line were found dead the next day after eggs were recorded. Similarly, Theologidis and colleagues (2014) found that survivorship differences did not explain decreased outcrossing rates in high salinity environment. Conceivably, the pattern observed in our study could have resulted from male and/or female gamete production failure. Indeed, it has been shown that increased temperature affects sperm and oocyte production, ovulation and spermatid activation in *C. elegans* (Aprison and Ruvinsky 2014; Petrella 2014). We might also expect thermal stress to result in elevated gamete death (McMullen et al. 2012) as increased temperature during ovulation has been shown to reduce gamete viability in some fish species (Pankhurst and Van Der Kraak 1997). However, low incidence of sterility in selfing lines proves that both types of gametes successfully function in hermaphrodites under the same thermal conditions. Hence, we hypothesize that the higher thermal sensitivity of obligatorily outcrossing lines may be associated with mating failure. Temperature is well-known to affect behavior of ectotherms (reviewed by Angiletta 2009) including reproductive behavior in many species (Wilkes 1963; Linn and Campbell 1988; Katsuki and Miyatake 2009). Whereas self-fertilization is a purely physiological process, outcrossing requires a complex set of behaviors in *C. elegans*. First, males have to respond to chemosensory cues from potential partners (Simon and Sternberg 2002). Then, they need to locate the vulva, to which they insert their spicules and ejaculate (Barr and Garcia 2006). Such a complex process is likely to be sensitive to environmental conditions, as disturbance at any of its components will result in reduced mating ability of an animal. We are not aware of any studies specifically addressing thermal effects on *C. elegans* mating behavior, however, it has been shown to be sensitive to intrinsic stress caused by senescence. Chatterjee et al. (2013) demonstrated that reproductive senescence in *C. elegans* males is associated with decreased mating efficiency rather than deterioration of sperm quality or sperm number.

Elucidating the mechanisms behind the pattern observed in our study requires further work. Whatever the mechanism, however, our results highlight the fact that the level of stress created by the same change in external environment (5 °C increase in ambient temperature) can differ dramatically between individuals differing in reproductive mode. Importantly, the sharp decline in mean fitness in outcrossing lines was also associated with an interesting pattern of variation: while 57% of pairs were unfertile and 30% only produced 1–6 larvae, four pairs (13%) bred 23, 30, 109 and 282 offspring, respectively. In hermaphrodites, such a heterogeneous response has been observed only under much more severe thermal stress (≥28 °C; Mc Mullen et al. 2012).

Such a pattern of response to a stressful environmental factor can have complex effects on adaptation process in outcrossing populations. On one hand, very low (or zero) fitness of most individuals translates to low effective population size, which increases the impact of genetic drift and may also lead to inbreeding depression, hampering adaptive potential and increasing the risk of extinction. On the other hand, large variation in fitness will generate strong selection on any traits associated with it, which can increase the rate of adaptation, as long as there is heritable variation in these traits (Lynch and Walsh 1998). For example, if mating efficiency does indeed strongly contribute to the reproductive performance of outcrossers, as we hypothesize, high temperature will impose strong selection on traits associated with mating success. This would further lead to intense sexual selection over mating, making sexual selection an important contributor to adaptation process in populations of outcrossers (Candolin and Heuschele 2008; Lorch et al. 2003; Plesnar-Bielak et al. 2012).

Importantly, we measured reproductive success of individual hermaphrodites or male–female pairs. Extrapolating these results to population level would make the estimated disadvantage of outcrossing even more severe: since in dioecious population only 50% of individuals can bear offspring (assuming 1:1 sex ratio). Thus, reproductive output of females should be at least twice that of hermaphrodites to offset the cost of males, whereas we showed it to be, on average, only about 1.5 times larger in 20 °C and 9.4–14.6 times smaller in 25 °C. However, in the population context, a small fraction of males may fertilize all or nearly all females. Thus, if the observed pattern of outcrossing pairs fitness at 25 °C was indeed caused by high incidence of male mating or fertilization failure, reproductive output of outcrossing populations in thermal stress conditions could be considerably higher than predicted from our pairs/individual based fecundity assays (but see Theologidis et al. 2014). Thus, determining population dynamics of different reproductive modes under stressful conditions needs experimental verification.

Summarizing, our results have important implications for investigating the evolution of reproductive modes in the context of environmental changes. They indicate that in addition to the level of genetic shuffling, reproductive modes may differ in the level of selective pressure experienced under the same external environment. Importantly, the difference in selective pressures, and its relative contribution to the adaptation process, may be specific to the nature of environmental change and the genetic make-up of evolving populations, among other putative factors. Thus, we suggest that future studies should test how hermaphrodite, male and female fitness is influenced by a variety of different stressors, using populations of various genetic backgrounds, including other *C. elegans* strains (although as shown by a comprehensive recent study, genetic diversity in *C. elegans* is exceptionally low in a global scale; Andersen et al. 2012) and other species, and also—other mechanisms determining reproductive mode (here, we applied two mutations most commonly used for this purpose in our model species, cf. Anderson et al. 2010). Most importantly, however, the potential for difference in selective pressures should be taken into account when assessing the role of outcrossing in adaptation process. Any differences in adaptation dynamics observed between reproductive modes should not be attributed solely, or primarily, to the effects of recombination, without checking for differences in selection.
